# Psychometric properties of the traditional Chinese version of the COVID Stress Scales in Hong Kong

**DOI:** 10.3389/fpubh.2023.1149221

**Published:** 2023-03-24

**Authors:** Ting Kin Ng, Wai Chan, Kitty Wan Ching Wang

**Affiliations:** ^1^Department of Psychology, Lingnan University, Tuen Mun, Hong Kong SAR, China; ^2^Department of Rehabilitation Sciences, The Hong Kong Polytechnic University, Kowloon, Hong Kong SAR, China; ^3^Department of Social and Behavioural Sciences, City University of Hong Kong, Kowloon, Hong Kong SAR, China

**Keywords:** COVID-19, stress, Hong Kong, validation study, factor analysis

## Abstract

**Introduction:**

The COVID Stress Scales (CSS) assess six domains of COVID-19-related stress, including (a) COVID danger, (b) COVID socioeconomic consequences, (c) COVID xenophobia, (d) COVID contamination, (e) COVID traumatic stress symptoms, and (f) COVID compulsive checking. Although the CSS have been validated in various cultural contexts, their psychometric properties in Hong Kong have not been examined. This study endeavors to validate the traditional Chinese version of the 36-item CSS (CSS-36) and the 18-item CSS (CSS-18) in Hong Kong.

**Method:**

Participants were 521 Hong Kong undergraduate students (61% female) aged from 18 to 26 years (*M* = 20.65, *SD* = 1.56). An online questionnaire was used for data collection.

**Results:**

The results of confirmatory factor analyses supported a six-factor structure for both the CSS-36 and the CSS-18. Multiple-group confirmatory factor analyses established the gender invariance of the six-factor model for both the CSS-36 and the CSS-18. The CSS-36 and the CSS-18 exhibited good internal consistency reliability and concurrent validity with fear of COVID-19 and negative emotional states.

**Discussion:**

The findings offer evidence for the psychometric properties of the traditional Chinese version of the CSS-36 and the CSS-18 in the Hong Kong context.

## Introduction

The Coronavirus disease 2019 (COVID-19) pandemic has exerted long-lasting impacts on multiple domains of health (1–3). The first COVID-19 case was identified in Wuhan, China in December 2019 (4, 5). This disease was later declared by the World Health Organization (6) as a pandemic on 11 March 2020.

On top of its detrimental impacts on physical health (1–3), the COVID-19 pandemic has also undermined people's mental health (7), and elevated levels of stress, fear, and anxiety were common in the general population during pandemics [e.g., (8–10)]. A systematic review of studies documented that stress was prevalent across countries during the COVID-19 pandemic (11). The rate of psychological distress rose up to 8 times since the outbreak (12). Stress related to COVID-19 has emerged as a major public health issue during the pandemic (8–11).

### COVID Stress Scales

Taylor et al. (13) developed the COVID-19 Stress Scales (CSS) to assess stress related to COVID-19. The CSS consist of 36 items measuring six domains of COVID-19-related stress: (a) fear of the danger of COVID-19 (COVID danger), (b) fear of the socioeconomic consequences of COVID-19 (COVID socioeconomic consequences), (c) fear that foreigners might carry COVID-19 (COVID xenophobia), (d) fear of sources of contamination related to COVID-19 (COVID contamination), (e) traumatic stress symptoms related to COVID-19 (COVID traumatic stress symptoms), and (f) compulsive checking and reassurance seeking related to COVID-19 (COVID compulsive checking). Most studies on the CSS have supported a six-factor structure (5, 14–18). Other studies have identified a five-factor structure, in which the items measuring COVID danger and COVID contamination were combined into a factor of COVID danger and contamination (13, 19, 20). The CSS have exhibited good concurrent validity with constructs such as fear of COVID-19 (16, 18, 21), depression, anxiety, stress (14, 16, 18), health anxiety, posttraumatic stress disorder symptoms, and compulsive washing (15).

The original English version of the CSS was first validated in the Canadian and American populations (13). Subsequently, the CSS have been translated into various languages and have demonstrated adequate psychometric properties in different cultural contexts such as Germany (15), Egypt (19), Iran (20), Mainland China (18), the Netherlands (21), Palestine (16), Poland (21), Saudi Arabia (19), Serbia (17), Spain (5), and Sweden (14). However, the psychometric properties of the CSS in Hong Kong have not been examined. Although Xia et al. (18) validated the simplified Chinese version of the CSS in Mainland China, simplified Chinese instruments may not be entirely applicable in the Hong Kong context because of linguistic and cultural differences (22, 23). While simplified Chinese is the official written language in Mainland China, traditional Chinese is the official written language in Hong Kong. Because of cultural and historical reasons, simplified Chinese and traditional Chinese have different writing systems and are not regarded as interchangeable (24). Moreover, there are cultural differences between Hong Kong and Mainland China, and Mainland Chinese generally perceive Hong Kong people as more Westernized than them (25, 26). Therefore, it is necessary to develop a traditional Chinese version of the CSS and investigate its psychometric properties in Hong Kong.

Besides, the gender invariance of the CSS has rarely been examined, although females tended to experience higher levels of fear and anxiety of COVID-19 (12). Nonetheless, group differences cannot be interpreted unambiguously without establishing factorial invariance (27). Hence, examining the gender invariance of the CSS is crucial for understanding gender differences in COVID-19-related stress. The study by Noe-Grijalva et al. (5) supported the gender invariance for the Spanish version of the CSS. It is of theoretical interest to examine whether the CSS are invariant across gender among people in Hong Kong.

One disadvantage of the original 36-item CSS (CSS-36) is that the scales are relatively lengthy (28). Thibault et al. (28) recently validated an 18-item CSS (CSS-18) in Canadian university students. This brief version provides researchers with an option to save time and lower participants' fatigue without compromising the psychometric properties. Further validation studies on the CSS-18 in other languages are needed.

### The current study

Hong Kong had low COVID-19 infection and death rates until the outbreak of the Omicron variant in early 2022. The high population density of Hong Kong has implied a very high contagion risk (29). The coping responses (e.g., panic buying) observed in Hong Kong residents are considered maladaptive and anxiety provoking (30), and might lead to an elevated level of stress among Hong Kong people. It is timely to understand COVID-19-related stress among Hong Kong people.

During the COVID-19 outbreak, students worldwide have experienced high levels of stress (11). Research has shown that younger age and student status were significant risk factors for greater distress during the COVID-19 pandemic (11). Students have experienced decreased wellbeing and increased emotional problems after the COVID-19 outbreak (31–33). More than one tenth of undergraduate and graduate students had high levels of COVID-19 fear and psychological symptoms but low levels of resilience (10). The COVID-19-related stress and mental health of students deserve special attention. However, the COVID-19-related stress of students has not been well studied in the context of Hong Kong and further research is needed.

The primary goal of the current study is to validate the traditional Chinese version of the CSS-36 (13) and the CSS-18 (28) in a sample of undergraduate students in Hong Kong. First, we examined the factor structure of the CSS. Specifically, the five-factor (13, 19, 20) and six-factor models (5, 14, 16, 17) identified in past studies were tested. Second, we investigated the factorial invariance of the CSS across gender. Third, we examined the internal consistency reliability of the CSS. Fourth, we investigated the concurrent validity of the CSS by investigating their relationships with fear of COVID-19 and negative emotional states (depression, anxiety, and stress), which are conceptually and empirically related to the CSS domains (16).

## Method

### Participants and procedure

Participants were 521 undergraduate students from a local university in Hong Kong. The data were collected in June 2022. Inclusion criteria included undergraduate students in Hong Kong and the ability to read traditional Chinese. Exclusion criteria included age under 18 years and the inability to provide informed consent. The participants were recruited from a local university in Hong Kong. All undergraduate students from the university were invited through a mass email to participate in the present study. An online questionnaire was used for data collection. Each eligible participant received a unique and personal link. Informed consent was obtained from the participants prior to their participation in the study. Participation was on a voluntary basis. Each participant received a monetary incentive of $50 HKD (~$6.4 USD) for completing the survey.

### Measures

#### COVID-19-related stress

The CSS (13) were used to measure the levels of COVID-19-related stress. Following Hambleton's (34) guidelines of test translation and adaptation, the original English items of the CSS were first translated into traditional Chinese by a translator and then back-translated to English by another translator to ensure conceptual equivalence and accuracy. The traditional Chinese items are presented in the Supplementary material Table 1. The CSS assess six domains of COVID-19-related stress, including (a) COVID danger, (b) COVID economic consequences, (c) COVID xenophobia, (d) COVID contamination, (e) COVID traumatic stress symptoms, and (f) COVID compulsive checking. Each domain is measured by 6 items in the CSS-36 (13) and by 3 items in the CSS-18 (28). Each item is rated on a 5-point scale ranging from 0 to 4.

#### Fear of COVID-19

Participants' fear of COVID-19 was assessed using the Fear of COVID-19 Scale [FCV-19S; (35)]. This instrument comprises seven items (e.g., “I am most afraid of coronavirus-19”). All items are rated on a 5-point scale ranging from 1 (*strongly disagree*) to 5 (*strongly agree*). This study adopted the Chinese verison of the FCV-19S validated by Chi et al. (36).

#### Negative emotional states

The 21-item Depression Anxiety Stress Scales [DASS-21; (37)] was used to measure participants' levels of negative emotional states. The DASS-21 consists of three subscales, including depression (seven items), anxiety (seven items), and stress (seven items). Each item is scored on a 4-point scale ranging from 0 (*did not apply to me at all*) to 3 (*applied to me very much*). This study employed the Chinese version of the DASS validated by Taouk et al. (38).

### Data analysis

To investigate the factor structure of the CSS-36 and the CSS-18 in Hong Kong, CFAs were conducted using LISREL 8.80. The five-factor and six-factor models identified in prior studies were examined. Besides, an alternative one-factor model in which all items were loaded on a general factor was also tested. The model fit was evaluated with a combination of fit indices. A RMSEA value <0.10 represents an acceptable fit and <0.08 represents a satisfactory fit (39). A CFI > 0.95 and a NNFI > 0.95 indicate an adequate model fit (40). The Akaike information criterion (AIC) was adopted to compare the goodness-of-fit of the models. The AIC can compare nested or non-nested model, and a smaller value reflects a superior model fit (41).

To examine the gender invariance of the six-factor models for the CSS-36 and the CSS-18 in Hong Kong, multiple-group CFAs were performed using LISREL 8.80. A series of hierarchical steps were taken to evaluate factorial invariance (27). To assess configural invariance, the factor model without cross-group equality constraints was estimated across the two gender groups. Subsequently, equality constraints were imposed on the factor loadings (metric invariance), item intercepts (scalar invariance), error variances (error variance invariance), factor variances (factor variance invariance), and factor covariances (factor covariance invariance) across the two gender groups. Concerning model comparison, as the chi-square difference test is sensitive to sample size and excessively stringent, the change in CFI (ΔCFI) was used to examine gender invariance (27). A decrease in CFI > 0.01 reflects a significant decline in the model fit (27).

## Results

### Demographic characteristics

Among the 521 participants, there were 203 males (39.0%) and 318 females (61.0%). Their age ranged from 18 to 26 years (*M* = 20.65, *SD* = 1.56). They were from different faculties including arts (*n* = 218, 41.8%), business (*n* = 156, 29.9%), social sciences (*n* = 144, 27.6%), and others (*n* = 3, 0.6%).

### Confirmatory factor analysis

Since the tests of multivariate skewness and kurtosis indicated that the data did not follow multivariate normality (*p*s < 0.001), the robust maximum likelihood (RML) estimation was employed, and the Satorra-Bentler scaled χ^2^ (S-Bχ^2^) statistics were calculated to adjust for the non-normal distribution.

Table 1 presents results of CFAs for the CSS-36. The one-factor model failed to achieve a good model fit. The five-factor model fitted the data reasonably well, S-Bχ^2^ (584, *N* = 521) = 2601.47, *p* < 0.001, RMSEA = 0.081, 90% CI [0.078, 0.085], CFI = 0.976, NNFI = 0.974, AIC = 2765.47. The six-factor model demonstrated a satisfactory model fit, S-Bχ^2^ (579, *N* = 521) = 2106.96, *p* < 0.001, RMSEA = 0.071, 90% CI [0.068, 0.075], CFI = 0.982, NNFI = 0.980, AIC = 2280.96. Moreover, the smallest AIC value was found for the six-factor model, indicating that it had the best model fit. The six-factor model was selected as the final model. As presented in Table 2, all standardized factor loadings were stronger than 0.30 (*p*s < 0.001), and significant correlations were found among the six factors (*r*s = 0.61 to 0.85, *p*s < 0.001).

**Table 1:** Confirmatory factor analyses.

**Model**	**S-Bχ^2^**	***df***	**RMSEA [90% CI]**	**CFI**	**NNFI**	**AIC**
**36-item COVID Stress Scales**						
1-factor model	7763.69^***^	594	0.152 [0.149, 0.155]	0.913	0.908	7907.69
5-factor model	2601.47^***^	584	0.082 [0.078, 0.085]	0.976	0.974	2765.47
6-factor model	2106.96^***^	579	0.071 [0.068, 0.075]	0.982	0.980	2280.96
**18-item COVID Stress Scales**						
1-factor model	1900.95^***^	135	0.159 [0.152, 0.165]	0.911	0.905	1972.95
5-factor model	552.65^***^	125	0.081 [0.074, 0.088]	0.979	0.974	644.65
6-factor model	421.99^***^	120	0.070 [0.062, 0.077]	0.985	0.981	523.99

*** p < 0.001.

**Table 2:** Factor loadings and correlations.

	**36-item COVID Stress Scales**						**18-item COVID Stress Scales**					
	**1**	**2**	**3**	**4**	**5**	**6**	**1**	**2**	**3**	**4**	**5**	**6**
Item 1	0.86						0.81					
Item 2	0.84											
Item 3	0.71											
Item 4	0.76						0.80					
Item 5	0.72						0.75					
Item 6	0.82											
Item 7		0.90						0.87				
Item 8		0.92										
Item 9		0.92										
Item 10		0.83										
Item 11		0.86						0.86				
Item 12		0.78						0.83				
Item 13			0.86						0.82			
Item 14			0.92									
Item 15			0.91						0.87			
Item 16			0.82									
Item 17			0.72						0.72			
Item 18			0.81									
Item 19				0.91						0.88		
Item 20				0.83						0.83		
Item 21				0.78						0.81		
Item 22				0.81								
Item 23				0.79								
Item 24				0.74								
Item 25					0.88						0.84	
Item 26					0.79							
Item 27					0.75							
Item 28					0.83						0.85	
Item 29					0.90						0.91	
Item 30					0.86							
Item 31						0.84						
Item 32						0.84						0.78
Item 33						0.83						
Item 34						0.71						
Item 35						0.68						0.81
Item 36						0.73						0.80
1. COVID D	-						-					
2. COVID SE	0.64	-					0.61	-				
3. COVID X	0.67	0.64	-				0.68	0.71	-			
4. COVID C	0.81	0.67	0.85	-			0.79	0.62	0.85	-		
5. COVID T	0.65	0.65	0.65	0.73	-		0.61	0.66	0.69	0.66	-	
6. COVID CH	0.63	0.61	0.61	0.68	0.80	-	0.59	0.66	0.66	0.62	0.83	-

D, danger; SE, socioeconomic consequences; X, xenophobia; C, contamination; T, traumatic stress symptoms; CH, compulsive checking. All loadings and factor correlations are significant at p < 0.001.

The results of CFAs for the CSS-18 are presented in Table 1. The one-factor model did not exhibit a good model fit. The five-factor model showed an acceptable model fit, S-Bχ^2^ (125, *N* = 521) = 552.65, *p* < 0.001, RMSEA = 0.081, 90% CI [0.074, 0.088], CFI = 0.979, NNFI = 0.974, AIC = 664.65. The six-factor model attained an adequate model fit, S-Bχ^2^ (120, *N* = 521) = 421.99, *p* < 0.001, RMSEA = 0.070, 90% CI [0.062, 0.077], CFI = 0.985, NNFI = 0.981, AIC = 523.99. The six-factor model had the smallest AIC value and was chosen as the final model (see Table 1). As shown in Table 2, all standardized factor loadings were >30 (*p*s < 0.001), and the six factors were significantly intercorrelated (*r*s = 0.59 to 0.85, *p*s < 0.001).

### Gender invariance

Because tests of multivariate skewness and kurtosis revealed that multivariate normality was not held for the data of males and females, the RML estimation was adopted and the S-Bχ^2^ statistics were computed.

The results of gender invariance tests are summarized in Table 3. For the CSS-36, the baseline model fitted the data well, S-Bχ^2^ (1,158, *N* = 521) = 2,741.87, *p* < 0.001, RMSEA = 0.073, 90% CI [0.069, 0.076], CFI = 0.981, NNFI = 0.979. Constraining the factor loadings (ΔCFI = 0.000), item intercepts (ΔCFI = −0.002), item error variances (ΔCFI = 0.000), factor variances (ΔCFI = 0.000), and factor covariances (ΔCFI = 0.000) to be equal across the two gender groups did not significantly reduce the model fit. The final model demonstrated a good model fit, S-Bχ^2^ (1,275, *N* = 521) = 3047.29, *p* < 0.001, RMSEA = 0.073, 90% CI [0.070, 0.077], CFI = 0.979, NNFI = 0.979. These results supported configural invariance, metric invariance, scalar invariance, error variance invariance, factor variance invariance, and factor covariance invariance across the two gender groups. The male group serves as the reference group, in which the factor means were set at zero. Hence, the factor means of the female group indicated the mean differences across the two groups. Females reported greater scores on the subscales of COVID xenophoia (*t* = 3.35, *p* < 0.001) and COVID contamination (*t* = 4.08, *p* < 0.001) than males did. No gender differences were found for the other subscale scores.

**Table 3:** Invariance across gender.

**Model**	**S-Bχ^2^**	***df***	**RMSEA [90% CI]**	**CFI**	**NNFI**	**Comparison**	**ΔCFI**
**36-item COVID Stress Scales**							
1. Configural invariance	2741.87^***^	1,158	0.073 [0.069, 0.076]	0.981	0.979		
2. Metric invariance	2793.02^***^	1,188	0.072 [0.069, 0.076]	0.981	0.979	2 vs. 1	0.000
3. Scalar invariance	2943.19^***^	1,218	0.074 [0.070, 0.077]	0.979	0.978	3 vs. 2	−0.002
4. Error variance invariance	2984.78^***^	1,254	0.073 [0.070, 0.076]	0.979	0.979	4 vs. 3	0.000
5. Factor variance invariance	2999.08^***^	1,260	0.073 [0.070, 0.076]	0.979	0.979	5 vs. 4	0.000
6. Factor covariance invariance	3047.29^***^	1,275	0.073 [0.070, 0.077]	0.979	0.979	6 vs. 5	0.000
**18-item COVID Stress Scales**							
1. Configural invariance	555.88^***^	240	0.071 [0.063, 0.079]	0.984	0.980		
2. Metric invariance	567.12^***^	252	0.069 [0.062, 0.077]	0.984	0.981	2 vs. 1	0.000
3. Scalar invariance	608.06^***^	264	0.071 [0.063, 0.078]	0.983	0.980	3 vs. 2	−0.001
4. Error variance invariance	636.46^***^	282	0.070 [0.062, 0.077]	0.982	0.981	4 vs. 3	−0.001
5. Factor variance invariance	645.11^***^	288	0.069 [0.062, 0.076]	0.982	0.981	5 vs. 4	0.000
6. Factor covariance invariance	654.61^***^	303	0.067 [0.060, 0.074]	0.982	0.982	6 vs. 5	0.000

*** *p* < 0.001.

For the CSS-18, a satisfactory fit was found for the baseline model, S-Bχ^2^ (240, *N* = 521) = 555.88, *p* < 0.001, RMSEA = 0.071, 90% CI [0.063, 0.079], CFI = 0.984, NNFI = 0.980. Imposing cross-group equality constraints on the factor loadings (ΔCFI = 0.000), item intercepts (ΔCFI = −0.002), item error variances (ΔCFI = 0.000), factor variances (ΔCFI = 0.000), and factor covariances (ΔCFI = 0.000) did not significantly worsen the model fit. The final model achieved an adequate model fit, S-Bχ^2^ (303, *N* = 521) = 654.61, *p* < 0.001, RMSEA = 0.067, 90% CI [0.060, 0.074], CFI = 0.982, NNFI = 0.982. Configural invariance, metric invariance, scalar invariance, error variance invariance, factor variance invariance, and factor covariance invariance were established across the two gender groups. The factor means of the reference group (male) were set at zero. The factor means of the female group revealed that females reported greater subscale scores of COVID danger (*t* = 2.07, *p* < 0.05), COVID xenophoia (*t* = 2.97, *p* < 0.01) and COVID contamination (*t* = 4.68, *p* < 0.001) compared with males. Gender differences were not observed for the other subscale scores.

### Concurrent validity

The descriptive statistics of the CSS-36 and the CSS18 are summarized in Table 4. To assess the concurrent validity of the CSS-36 and the CSS-18 in Hong Kong, the relationships of the CSS-36 and the CSS-18 with fear of COVID-19 and negative emotional states (depression, anxiety, and stress) were investigated. As indicated in Table 5, the measures of fear of COVID-19 and negative emotional states had adequate internal consistency reliability (Cronbach's αs = 0.85 to 0.95, McDonald's ω = 0.85 to 0.95). For the CSS-36, the overall and subscales scores had strong correlations with fear of COVID-19 (*r*s = 0.52 to 0.75, *p*s < 0.001) and smaller but significant correlations with depression (*r*s = 0.12 to 0.23, *p*s < 0.01), anxiety (*r*s = 0.21 to 0.31, *p*s < 0.001), stress (*r*s = 0.18 to 0.26, *p*s < 0.001), and the overall DASS-21 score (*r*s = 0.18 to 0.29, *p*s < 0.001). Similarly, the overall and subscale scores of the CSS-18 also showed strong correlations with fear of COVID-19 (*r*s = 0.50 to 0.75, *p*s < 0.001) and smaller but significant correlations with depression (*r*s = 0.10 to 0.20, *p*s < 0.05), anxiety (*r*s = 0.21 to 0.30, *p*s < 0.001), stress (*r*s = 0.17 to 0.23, *p*s < 0.001), and the overall DASS-21 score (*r*s = 0.18 to 0.27, *p*s < 0.001). In sum, the concurrent validity of the CSS-36 and the CSS-18 among Hong Kong undergraduate students was supported.

**Table 4:** Descriptive statistics and correlations of the COVID Stress Scales.

	**1**	**2**	**3**	**4**	**5**	**6**	**7**
**36-item COVID Stress Scales**							
1. COVID danger							
2. COVID socioeconomic consequences	0.59^***^						
3. COVID xenophobia	0.62^***^	0.61^***^					
4. COVID contamination	0.73^***^	0.64^***^	0.79^***^				
5. COVID traumatic stress symptoms	0.59^***^	0.61^***^	0.61^***^	0.67^***^			
6. COVID compulsive checking	0.57^***^	0.57^***^	0.57^***^	0.62^***^	0.74^***^		
7. Overall COVID Stress Scales	0.82^***^	0.81^***^	0.85^***^	0.89^***^	0.83^***^	0.80^***^	
*M*	1.48	0.80	1.02	1.13	0.54	0.89	0.97
*SD*	0.88	0.91	0.90	0.86	0.70	0.76	0.70
Cronbach's α	0.91	0.95	0.94	0.92	0.93	0.90	0.97
McDonald's ω	0.91	0.95	0.94	0.92	0.93	0.90	0.97
**18-item COVID Stress Scales**							
1. COVID danger							
2. COVID socioeconomic consequences	0.52^***^						
3. COVID xenophobia	0.57^***^	0.62^***^					
4. COVID contamination	0.68^***^	0.54^***^	0.73^***^				
5. COVID traumatic stress symptoms	0.53^***^	0.60^***^	0.61^***^	0.59^***^			
6. COVID compulsive checking	0.49^***^	0.57^***^	0.56^***^	0.53^***^	0.72^***^		
7. Overall COVID Stress Scales	0.79^***^	0.80^***^	0.85^***^	0.85^***^	0.81^***^	0.78^***^	
*M*	1.56	0.83	1.04	1.37	0.50	0.70	1.00
*SD*	0.93	0.94	0.91	0.93	0.72	0.76	0.70
Cronbach's α	0.83	0.89	0.84	0.88	0.89	0.84	0.94
McDonald's ω	0.85	0.89	0.85	0.88	0.90	0.84	0.94

*** p < 0.001.

**Table 5:** Correlations of COVID Stress Scales with fear of COVID-19 and negative emotional states.

	**FCV-19S**	**Depression**	**Anxiety**	**Stress**	**Overall DASS-21**
*M*	2.08	0.90	0.76	1.07	0.91
*SD*	0.81	0.72	0.63	0.70	0.63
Cronbach's α	0.90	0.89	0.85	0.87	0.95
McDonald's ω	0.91	0.89	0.85	0.87	0.95
**36-item COVID Stress Scales**					
1. COVID danger	0.64^***^	0.15^***^	0.26^***^	0.19^***^	0.22^***^
2. COVID socioeconomic consequences	0.52^***^	0.19^***^	0.27^***^	0.20^***^	0.23^***^
3. COVID xenophobia	0.62^***^	0.12^**^	0.21^***^	0.18^***^	0.18^***^
4. COVID contamination	0.67^***^	0.13^**^	0.25^***^	0.20^***^	0.21^***^
5. COVID traumatic stress symptoms	0.67^***^	0.23^***^	0.33^***^	0.26^***^	0.29^***^
6. COVID compulsive checking	0.66^***^	0.15^***^	0.25^***^	0.19^***^	0.21^***^
7. Overall COVID Stress Scales	0.75^***^	0.19^***^	0.31^***^	0.24^***^	0.26^***^
**18-item COVID Stress Scales**					
1. COVID danger	0.61^***^	0.15^***^	0.25^***^	0.19^***^	0.21^***^
2. COVID socioeconomic consequences	0.50^***^	0.17^***^	0.26^***^	0.19^***^	0.22^***^
3. COVID xenophobia	0.62^***^	0.12^**^	0.21^***^	0.17^***^	0.18^***^
4. COVID contamination	0.65^***^	0.10^*^	0.21^***^	0.19^***^	0.18^***^
5. COVID traumatic stress symptoms	0.66^***^	0.20^***^	0.31^***^	0.23^***^	0.27^***^
6. COVID compulsive checking	0.62^***^	0.15^***^	0.26^***^	0.18^***^	0.21^***^
7. Overall COVID Stress Scales	0.75^***^	0.18^***^	0.30^***^	0.23^***^	0.26^***^

FCV-19S, Fear of COVID-19 Scale; DASS-21, The 21-item Depression Anxiety Stress Scales.

* p < 0.05.

** p < 0.01.

*** p < 0.001.

### Internal consistency reliability

The reliability coefficients are summarized in Table 4. Regarding the CSS-36, the overall score (Cronbach's α = 0.97, McDonald's ω = 0.97) and all subscale scores (Cronbach's αs = 0.90 to 0.95, McDonald's ω = 0.90 to 0.95) yielded high reliability coefficients. Besides, the overall CSS-18 score (Cronbach's α = 0.94, McDonald's ω = 0.94) and all subscale scores (Cronbach's αs = 0.83 to 0.89, McDonald's ω = 0.84 to 0.90) also produced high reliability coefficients. Taken together, the CSS-36 and CSS-18 exhibited good internal consistency reliability among Hong Kong undergraduate students.

## Discussion

This study attempts to validate the CSS-36 and the CSS-18 in Hong Kong. It was found that the six-factor model demonstrated the best fit. The factorial invariance across gender was established. Females showed higher levels of COVID-19-related stress in the domains of COVID xenophobia and COVID contamination. The CSS showed good internal consistency reliability and concurrent validity with fear of COVID-19 and negative emotional states. This study provided initial evidence for the psychometric properties of the traditional Chinese version of the CSS-36 and the CSS-18 in a sample of Hong Kong undergraduate students.

Taylor et al.'s (13) theoretical framework of COVID-19-related stress encompasses six domains, including fear of the danger of being infected, fear of the socioeconomic consequences of the pandemic, fear that foreigners is spreading the virus, fear of contacting contaminated objects, traumatic stress symptoms regarding the pandemic, and compulsive checking and reassurance seeking concerning the pandemic. Consistent with prior studies (5, 14–18), this study supported the six-factor model for both the CSS-36 and the CSS-18 in Hong Kong. Although Taylor et al. (13) proposed six domains of COVID-19-related stress, their study identified a five-factor solution, which was supported by some other studies (19, 20). One possible explanation is that Taylor et al. (13) used the exploratory factor analysis to identify a five-factor solution, but did not compare it with a six-factor model. Studies examining both models have consistently favored the six-factor model over the five-factor model (5, 14, 17, 21). Similarly, this study found that the six-factor model fitted the data better than did the five-factor model for both the CSS-36 and the CSS-18. These findings resonate with the stress and coping model (42, 43). In particular, the COVID-19 pandemic is a stressor that may affect people's reactions and behavior in various ways (9, 30). Reducing the factor structure may result in information loss and an oversimplification of the domains of COVID-19-related stress.

Moreover, this study established the gender invariance of the six-factor model for both the CSS-36 and the CSS-18. These findings indicate that the items have the same meanings for males and females in Hong Kong, allowing meaningful comparisons of the six domains of COVID-19-related stress across gender in the Hong Kong context. This study found higher COVID xenophobia and COVID contamination in females compared with males. This echoed past research findings that females tend to experience higher levels of stress and negative emotions during the pandemic (44). This might be due to the traditional gender role of women (45). Females are often assumed to be caregivers in the family (46–48), resulting in gender inequalities during the COVID-19 outbreak (49). Compared with males, females are more likely to engage in domestic tasks such as household sterilization, grocery shopping, and caregiving for sick family members, and these tasks might be more challenging during the pandemic (49). The reasons for the gender differences in COVID-19-related stress deserve further investigations. Interestingly, similar to prior research (44), this study did not find a gender difference in the socioeconomic consequences of the pandemic. These findings suggest that higher COVID-19-related stress among females may be domain specific.

This study found that the CSS-36 and the CSS-18 showed comparable levels of reliability and validity in Hong Kong. These findings strengthen the confidence of applying the brief version of the CSS to wider contexts. The CSS-18 allow for a quicker assessment of the COVID-19-related stress (28) without compromising the psychometric properties. Future research is recommended to validate the CSS-18 in other cultural contexts. More important, the CSS may serve as a useful tool for assessing stress related to post-COVID conditions [i.e., long COVID; (50)]. Given that the COVID-19 pandemic has persisted for several years, it remains uncertain whether or not COVID-19-related stress will translate to chronic stress (51). Further studies may adopt the CSS to assess stress related to post-COVID conditions.

The findings of the current study have important practical implications. This study showed that the six domains of COVID-19-related stress (30) are applicable to Hong Kong undergraduate students. In this light, interventions for improving students' wellbeing during the pandemic may target some domains of COVID-19-related stress. For instance, interventions that aims at reducing compulsive checking may be particularly effective in improving mental health during the pandemic. Future studies are needed to examine the effectiveness of those interventions, and the CSS can be used to evaluate the treatment efficacy.

### Limitations and future research directions

There are several caveats in this study. First, this study used a cross-sectional design. Future longitudinal research is needed to examine additional psychometric properties of the of the CSS in Hong Kong, including test-retest reliability, longitudinal invariance, and predictive validity with later outcomes. Second, the current sample included undergraduate students in Hong Kong only. Further studies are required to verify the psychometric properties of the CSS in other age groups. Third, the study only used self-report measures to examine the validity of the CSS in Hong Kong. Future work may use neural or physiological measures of stress (e.g., salivary cortisol) to validate the CSS.

### Conclusions

The COVID-19 pandemic has caused stress and dampened the mental health of people worldwide. A psychometrically sound tool for assessing COVID-19-related stress is crucial for understanding the detrimental impacts of the COVID-19 pandemic on mental health and evaluating the efficacy of stress reduction intervention programs during the pandemic. To address the research needs, this study sought to evaluate the psychometric properties of the traditional Chinese version of the CSS among Hong Kong undergraduate students. The present results supported the six-factor structure of the CSS. The gender invariance of the six-factor model was established. Adequate internal consistency and concurrent validity were also achieved. The brief version of the CSS might provide a useful tool for more efficient assessments. Besides, the CSS could be useful for assessing stress pertinent to post-COVID-19 syndrome. The implications of the CSS are documented in this article. Future research is suggested to further validate the traditional Chinese version of the CSS in other age groups. More important, the conceptual framework and the factor structure of the CSS could serve as a blueprint for measures of stress related to future pandemics.

## Data availability statement

The raw data supporting the conclusions of this article will be made available by the authors, without undue reservation.

## Ethics statement

The studies involving human participants were reviewed and approved by Office of Research and Knowledge Transfer, Lingnan University. The patients/participants provided their written informed consent to participate in this study.

## Author contributions

TN designed the study and performed the data analysis. TN, WC, and KW conducted the literature review and drafted and revised the manuscript. All authors approved the submitted version of the manuscript.
