# Supplementary material for: Psychometric properties of the traditional Chinese version of the COVID Stress Scales in Hong Kong
Source: Front Public Health. 2023 Mar 24;11:1149221. doi: 10.3389/fpubh.2023.1149221 (PMC10080101; doi:10.3389/fpubh.2023.1149221)
Supplement: Supplementary file 1 [file Table_1.docx]

Appendix

*Items of the Traditional Chinese Version of the COVID Stress Scales*

| 以下題目關於你在過去七天中可能經歷的各種憂慮。在以下句子中，我們將2019冠狀病毒病稱為「病毒」。  The following asks about various kinds of worries that you might have experienced over the past seven days. In the following statements, we refer to COVID-19 as "the virus".   1. 我擔心感染病毒。   I am worried about catching the virus.   1. 我擔心基本的衛生習慣（例如洗手）不足以令我遠離病毒。   I am worried that basic hygiene (e.g., handwashing) is not enough to keep me safe from the virus.   1. 我擔心我們的醫療保健系統無法令我遠離病毒。   I am worried that our healthcare system is unable to keep me safe from the virus.   1. 我擔心我不能令我的家人遠離病毒。   I am worried that I can’t keep my family safe from the virus.   1. 我擔心我們的醫療保健系統無法保護我所愛的人。   I am worried that our healthcare system won’t be able to protect my loved ones.   1. 我擔心社交距離不足以讓我遠離病毒。   I am worried that social distancing is not enough to keep me safe from the virus.   1. 我擔心雜貨店的食物會賣光。   I am worried about grocery stores running out of food.   1. 我擔心雜貨店的感冒或流感藥物會賣光。   I am worried about grocery stores running out of cold or flu remedies.   1. 我擔心藥房的處方藥物會賣光。   I am worried about pharmacies running out of prescription medicines.   1. 我擔心雜貨店的飲用水會賣光。   I am worried about grocery stores running out of water.   1. 我擔心雜貨店的清潔或消毒用品會賣光。   I am worried about grocery stores running out of cleaning or disinfectant supplies.   1. 我擔心雜貨店會停業。   I am worried that grocery stores will close down.   1. 我擔心外來人在我的地區傳播病毒。   I am worried that foreigners are spreading the virus in my country.   1. 如果我遇到一個外來人，我會擔心他們可能帶有病毒。   If I met a person from a foreign country, I’d be worried that they might have the virus.   1. 我擔心與外來人接觸，因為他們可能帶有病毒。   I am worried about coming into contact with foreigners because they might have the virus.   1. 我擔心外來人會傳播病毒，因為他們不像我們那麼清潔。   I am worried that foreigners are spreading the virus because they’re not as clean as we are.   1. 如果我去一家專門經營外地食品的餐館，我會擔心感染病毒。   If I went to a restaurant that specialized in foreign foods, I’d be worried about catching the virus.   1. 如果我和一群外來人在升降機裏，我會擔心他們已感染了病毒。   If I was in an elevator with a group of foreigners, I’d be worried that they’re infected with the virus.   1. 我擔心我周圍的人會傳染病毒給我。   I am worried that people around me will infect me with the virus.   1. 我擔心如果我在公共場所觸摸某些東西（例如扶手、門柄），我會感染病毒。   I am worried that if I touched something in a public space (e.g., handrail, door handle), I would catch the virus.   1. 我擔心如果有人在我附近咳嗽或打噴嚏，我會感染病毒。   I am worried that if someone coughed or sneezed near me, I would catch the virus.   1. 我擔心我可能因為處理金錢或使用提款機而感染病毒。   I am worried that I might catch the virus from handling money or using a debit machine.   1. 我擔心在現金交易時取回零錢。   I am worried about taking change in cash transactions.   1. 我擔心我的郵件被郵件處理人員污染。   I am worried that my mail has been contaminated by mail handlers.  在以下句子中，我們將2019冠狀病毒病稱為「病毒」。請閱讀每個句子，並回答在過去七天中每個問題有多常出現。  In the following statements, we refer to COVID-19 as "the virus". Please read each statement and indicate how frequently each problem has been for you during the past seven days.   1. 我因為擔心病毒而睡得不好。   I had trouble sleeping because I worried about the virus.   1. 我做了關於病毒的惡夢。   I had bad dreams about the virus.   1. 我會無意中想起病毒。   I thought about the virus when I didn’t mean to.   1. 關於病毒的不安影像不由自主地出現在我的腦海裏。   Disturbing mental images about the virus popped into my mind against my will.   1. 我難以集中精神因為我一直想著病毒。   I had trouble concentrating because I kept thinking about the virus.   1. 令人想起病毒的事物使我有身體反應，例如出汗或心跳加速。   Reminders of the virus caused me to have physical reactions, such as sweating or a pounding heart.  以下問題關於檢查行為。在過去七天中，你有多少次因為擔心2019冠狀病毒病而檢查以下內容？  The following items ask about checking behaviours. During the past seven days, how much have you checked the following because of concerns about COVID-19?   1. 關於2019冠狀病毒病的社交媒體貼文。   Social media posts concerning COVID-19.   1. 關於2019冠狀病毒病的YouTube影片。   YouTube videos about COVID-19   1. 就2019冠狀病毒病的資訊向朋友或家人求證。   Seeking reassurance from friends or family about COVID-19.   1. 檢查你自己的身體是否有感染跡象（例如量度體溫）。   Checking your own body for signs of infection (e.g., taking your temperature).   1. 向醫療專業人士（例如醫生或藥劑師）尋求有關2019冠狀病毒病的建議。   Asking health professionals (e.g., doctors or pharmacists) for advice about COVID-19.   1. 在互聯網上搜尋 2019冠狀病毒病的治療方法。   Searched the Internet for treatments for COVID-19. |
| --- |
